# The Amelioration of Renal Damage in Skp2-Deficient Mice Canceled by p27 *^Kip1^* Deficiency in Skp2^−/−^ p27^−/−^ Mice

**DOI:** 10.1371/journal.pone.0036249

**Published:** 2012-04-27

**Authors:** Sayuri Suzuki, Hirotaka Fukasawa, Taro Misaki, Akashi Togawa, Naro Ohashi, Kyoko Kitagawa, Yojiro Kotake, Ning Liu, Hiroyuki Niida, Keiko Nakayama, Keiichi I. Nakayama, Tatsuo Yamamoto, Masatoshi Kitagawa

**Affiliations:** 1 Department of Molecular Biology, Hamamatsu University School of Medicine, Hamamatsu, Japan; 2 First Department of Medicine, Hamamatsu University School of Medicine, Hamamatsu, Japan; 3 Division of Developmental Genetics, Center for Translational and Advanced Animal Research on Human Diseases, Tohoku University Graduate School of Medicine, Sendai, Japan; 4 Department of Molecular and Cellular Biology, Medical Institute of Bioregulation, Kyushu University, Fukuoka, Japan; 5 Second Department of Medicine, Numazu City Hospital, Numazu, Japan; University of Minnesota, United States of America

## Abstract

SCF-Skp2 E3 ubiquitin ligase (Skp2 hereafter) targets several cell cycle regulatory proteins for degradation via the ubiquitin-dependent pathway. However, the target-specific physiological functions of Skp2 have not been fully elucidated in kidney diseases. We previously reported an increase in Skp2 in progressive nephropathy and amelioration of unilateral ureteral obstruction (UUO) renal injury associated with renal accumulation of p27 in Skp2^−/−^ mice. However, it remains unclear whether the amelioration of renal injury in Skp2^−/−^ mice is solely caused by p27 accumulation, since Skp2 targets several other proteins. Using Skp2^−/−^p27^−/−^ mice, we investigated whether Skp2 specifically targets p27 in the progressive nephropathy mediated by UUO. In contrast to the marked suppression of UUO renal injury in Skp2^−/−^ mice, progression of tubular dilatation associated with tubular epithelial cell proliferation and tubulointerstitial fibrosis with increased expression of collagen and α-smooth muscle actin were observed in the obstructed kidneys in Skp2^−/−^p27^−/−^ mice. No significant increases in other Skp2 target proteins including p57, p130, TOB1, cyclin A and cyclin D1 were noted in the UUO kidney in Skp2^−/−^ mice, while p21, c-Myc, b-Myb and cyclin E were slightly increased. Contrary to the ameliorated UUO renal injure by Skp2-deficiency, the amelioration was canceled by the additional p27-deficiency in Skp2^−/−^p27^−/−^ mice. These findings suggest a pathogenic role of the reduction in p27 targeted by Skp2 in the progression of nephropathy in UUO mice.

## Introduction

Cell proliferation is a basic biological mechanism that is controlled by a network of proteins including cyclins, cyclin-dependent kinases (CDKs) [1] and cyclin-dependent kinase inhibitors (CKIs) [2]. The CKI p27*^Kip1^* (p27), a known negative regulator of the cell cycle, is abundantly expressed in most normal quiescent cells, and its level declines when cells are stimulated to proliferate in response to mitotic stimuli [3], [4]. *In vitro* studies have shown that experimentally reducing the level of p27 protein augments the proliferative response to mitogens [5], [6], while forced overexpression of p27 inhibits cell proliferation [4]. The level of p27 protein is controlled not only by transcriptional activation but also by proteolytic degradation of p27 protein via the ubiquitin-proteasome system as a post-translational regulation. Consequently, G1-cyclin-CDK complexes become activated to phosphorylate retinoblastoma protein and advance the cell cycle from G1 to S phase [7], [8].

The ubiquitin-proteasome pathway of protein degradation plays an important role in controlling the abundance of cell cycle regulatory proteins [9], [10]. The rapidity and substrate specificity of protein degradation through the ubiquitin-proteasome pathway are consistent with its role in controlling the fluctuations in the intracellular concentrations of cyclins and CKIs. Skp2 is known to be the F-box protein component of an SCF-type ubiquitin ligase that interacts with p27, and the SCF-Skp2 complex promotes p27 degradation by ubiquitination [11], [12]. Skp2^−/−^ mice have been reported to show cellular accumulation of p27 [13]. Moreover, cdc kinase subunit 1 (Cks1) is required for degradation of p27 mediated by Skp2 [14]. It has also been reported that Skp2 targets several cell cycle regulatory proteins including p27, p21, p57, cyclin E, cyclin A and cyclin D1 for degradation via the ubiquitin-dependent pathway [15]. However, it remains unclear which proteins are targeted by Skp2 for degradation in specific biological processes or diseases.

In the kidney, cell proliferation is supposed to be a pivotal response to damage, and culminates in the development of renal injury and fibrosis. Proliferation of tubular cells is a characteristic feature of obstructed kidneys in unilateral ureteral obstruction (UUO). UUO is a representative model of progressive tubulointerstitial injury that is suitable for investigating the cellular and molecular events that occur during the progression of renal fibrosis associated with cell proliferation and apoptosis [16], [17]. An imbalance between cell proliferation and apoptosis has been shown to lead to unchecked apoptosis, resulting in progressive cell loss, renal tubular atrophy and interstitial fibrosis [18].

It has been reported that both the mRNA and protein levels of the CDKIs p27 and p21 are upregulated at an early stage in the obstructed kidneys of UUO mice [19]–[21]. Marked increases in renal tubular epithelial cell proliferation and apoptosis are also observed in the obstructed kidneys in p27^−/−^ mice [22]. Since upregulation of p27 safeguards against excessive renal epithelial cell proliferation, p27 may be involved in protecting cells and tissues against inflammatory injury. On the other hand, no significant changes in tubular epithelial cell proliferation and apoptosis are found in the obstructed kidneys in p21^−/−^ mice, despite the proliferation of interstitial cells, especially myofibroblasts [23]. Unlike p27, p21 limits the magnitude of early myofibroblast proliferation, but does not seem to be essential for the regulation of tubular epithelial cell proliferation and apoptosis following UUO [23]. These studies suggest differential regulatory roles for the CDKIs p27 and p21 in UUO kidneys.

Recently, we reported that Skp2 mRNA was increased in UUO kidneys and that the progression of fibrotic tubulointerstitial damage in UUO kidneys was attenuated in Skp2^−/−^ mice [24]. Although the p27 protein level was increased in the obstructed kidneys in wild-type (WT) mice, it was significantly higher in Skp2^−/−^ mice. p27 accumulation, which results from SCF-Skp2 ubiquitin ligase deficiency in Skp2^−/−^ mice, inhibited the renal tubular epithelial cell proliferation, and was involved to the amelioration of the renal damage induced by obstructive nephropathy. Moreover, we found upregulation of not only Skp2, but also Cks1, an essential cofactor for the SCF-Skp2 ubiquitin ligase in targeting p27, which were induced by activation of the TNF-α/NF-κB pathway in two models of chronic progressive nephropathy, namely UUO mice and chronic anti-thymocyte serum nephropathy rats [25]. However, because Skp2 has multiple targets including p21, p57, c-Myc, p130 and TOB1 in addition to p27 [26]–[30], little is known about the specific target of Skp2 in renal lesions.

In the present study, we investigated whether degradation of p27 targeted by Skp2 is required for the development of tubulointerstitial injury in UUO kidneys by comparing WT, Skp2^−/−^ and p27^−/−^ mice with Skp2^−/−^p27^−/−^ mice. Contrary to the amelioration of UUO renal injury by Skp2-deficiency, the amelioration was abolished by the additional deficiency of p27 in Skp2^−/−^p27^−/−^ double knockout mice. These findings suggest that p27 is the key molecule targeted by Skp2 that is involved in the progression of renal injury in UUO mice.

## Results

### Levels of Skp2 target proteins in the UUO kidney

**Figure 1:**
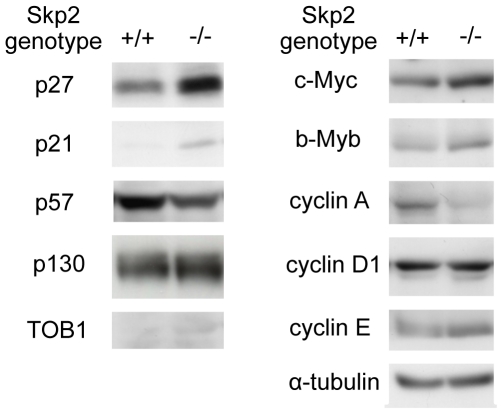
Immunoblot analyses of the previously reported Skp2 target proteins in UUO kidneys. The levels of the previously reported Skp2 target proteins were detected by western blot analysis in the obstructed kidneys in WT (Skp2^+/+^) and Skp2^−/−^ mice at 7 days after UUO. α-tubulin was evaluated as an internal control.

First, we investigated the levels of Skp2 target proteins in UUO renal injury in WT and Skp2^−/−^ mice. Consistent with our previous report [24], accumulation of p27 and p21 was observed in the UUO kidneys in Skp2^−/−^ mice (Figure 1). The levels of c-Myc, b-Myb and cyclin E were also slightly increased, whereas p57, p130, TOB1, cyclin A and cyclin D1 did not accumulate, in the UUO kidneys in Skp2^−/−^ mice. These findings suggest that the increased Skp2 promoted the degradation of p27, p21, c-Myc, b-Myb and cyclin E in the UUO kidneys. However, it remains unclear which protein degradation targeted by Skp2 plays an important role in the progression of the obstructive nephropathy. To address this question, we first focused on p27 and compared its levels in UUO renal injury in Skp2^−/−^p27^−/−^ double-knockout mice with those in WT, Skp2^−/−^ and p27^−/−^ mice.

### Genotypes of Skp2^−/−^, Skp2^−/−^p27^−/−^ and p27^−/−^ mice

The genotypes of the Skp2^−/−^, Skp2^−/−^p27^−/−^ and p27^−/−^ mice were confirmed by PCR (Figure S1A). p27 level was increased in Skp2^−/−^ mice comparing with wild-type mice after UUO operation, whereas p27 protein was not detected in both p27^−/−^ and Skp2^−/−^p27^−/−^ mice (Figure S1B).

### Diminished ameliorative effect of Skp2-deficiency on the obstructive renal injury in Skp2^−/−^p27^−/−^ mice

**Figure 2:**
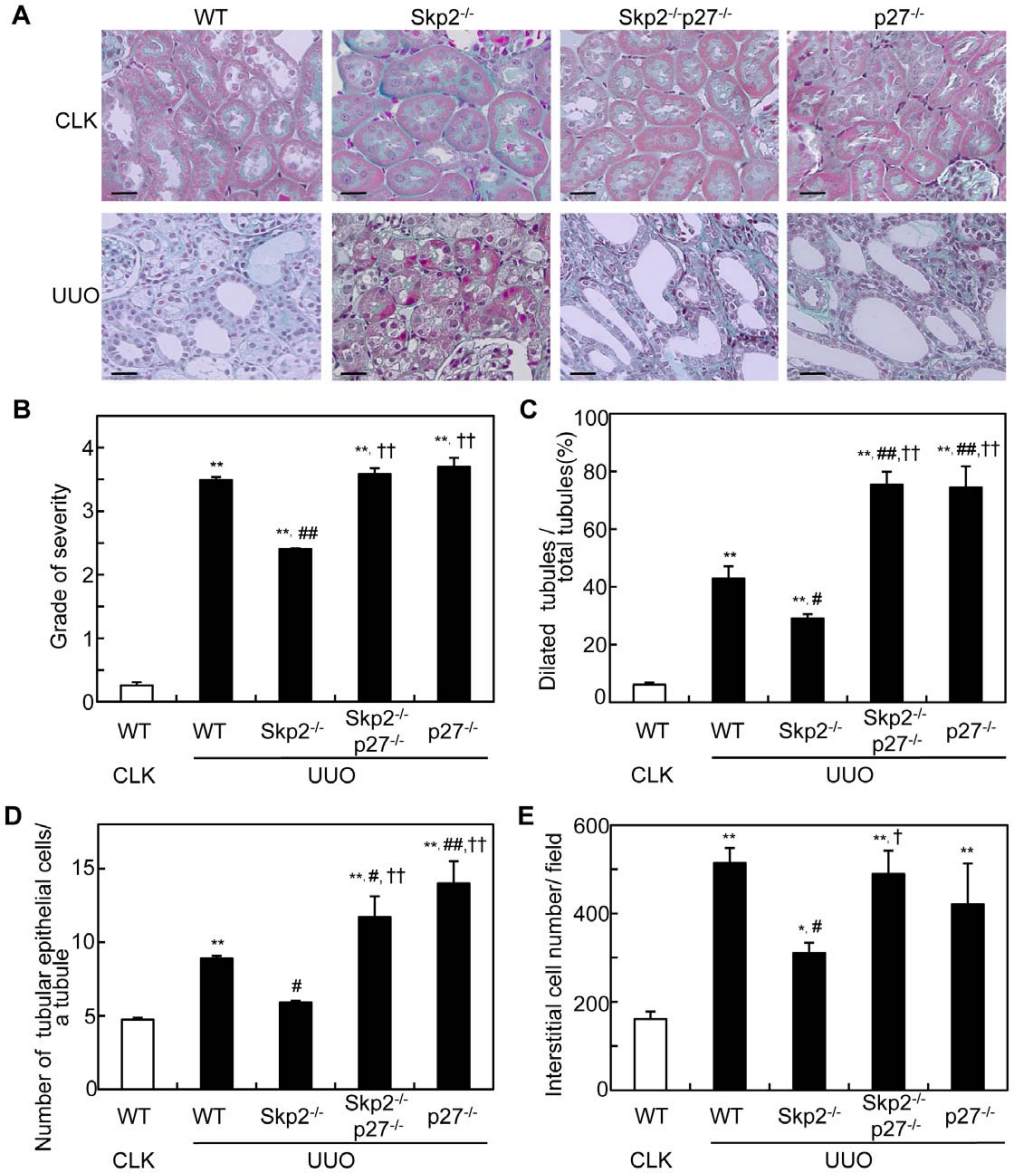
Levels of UUO renal injury. (**A**) Representative microscopic images of CLK and UUO kidneys in WT, Skp2^−/−^, Skp2^−/−^p27^−/−^ and p27^−/−^ mice (Masson's trichrome staining; scale bars: 50 µm). Increases in the interstitial area, tubular dilatation and atrophy, and interstitial cell infiltration are observed in the UUO kidneys in WT mice. However, the severities of these lesions are markedly less in the UUO kidneys in Skp2**^−/−^** mice. In contrast, aggravation of the UUO renal injury is noted in Skp2^−/−^p27^−/−^ and p27^−/−^ mice. (**B**) The severity of fibrotic tubulointerstitial lesions was graded semiquantitatively as follows: 0, absent (0%); 1, weak (≤10%); 2, mild (>10 to ≤30%); 3, moderate (>30 to ≤50%); 4, strong (>50%) in WT, Skp2^−/−^, Skp2^−/−^p27^−/−^ and p27^−/−^ mice at 7 days after UUO. (**C–E**) The numbers of dilated tubules (**C**), renal tubular epithelial cells in a tubule (**D**), and tubular interstitial cells (**E**) were counted and evaluated statistically in the UUO kidneys in WT, Skp2^−/−^, Skp2^−/−^p27^−/−^ and p27^−/−^ mice at 7 days after UUO. The CLK kidneys in WT mice were evaluated as controls. *P<0.05, **P<0.01 versus WT CLK kidneys, ^#^ *P*<0.05, ^##^ *P*<0.01 versus WT UUO kidneys and ^†^ *P*<0.05, ^††^ *P*<0.01 versus Skp2^−/−^ UUO kidneys.

In accordance with our previous report [24], remarkable amelioration of the tubulointerstitial fibrosis and significant decreases in the numbers of dilated tubules, tubular cells and interstitial cells were noted in the UUO kidneys in Skp2^−/−^ mice compared with WT mice. In contrast, the amelioration of the UUO renal injury noted in Skp2^−/−^ mice was almost completely abolished, and instead rather aggravated, by the additional p27-deficiency in Skp2^−/−^p27^−/−^ mice (Figure 2, A–E). Aggravation of the UUO renal injury was also observed in p27^−/−^ mice. There were no significant renal histological changes in the non-obstructed CLK kidneys in all genotypes of mice.

### Diminished suppressive effect of Skp2-deficiency on tubular epithelial cell proliferation and apoptosis in UUO kidneys in Skp2^−/−^p27^−/−^ mice

**Figure 3:**
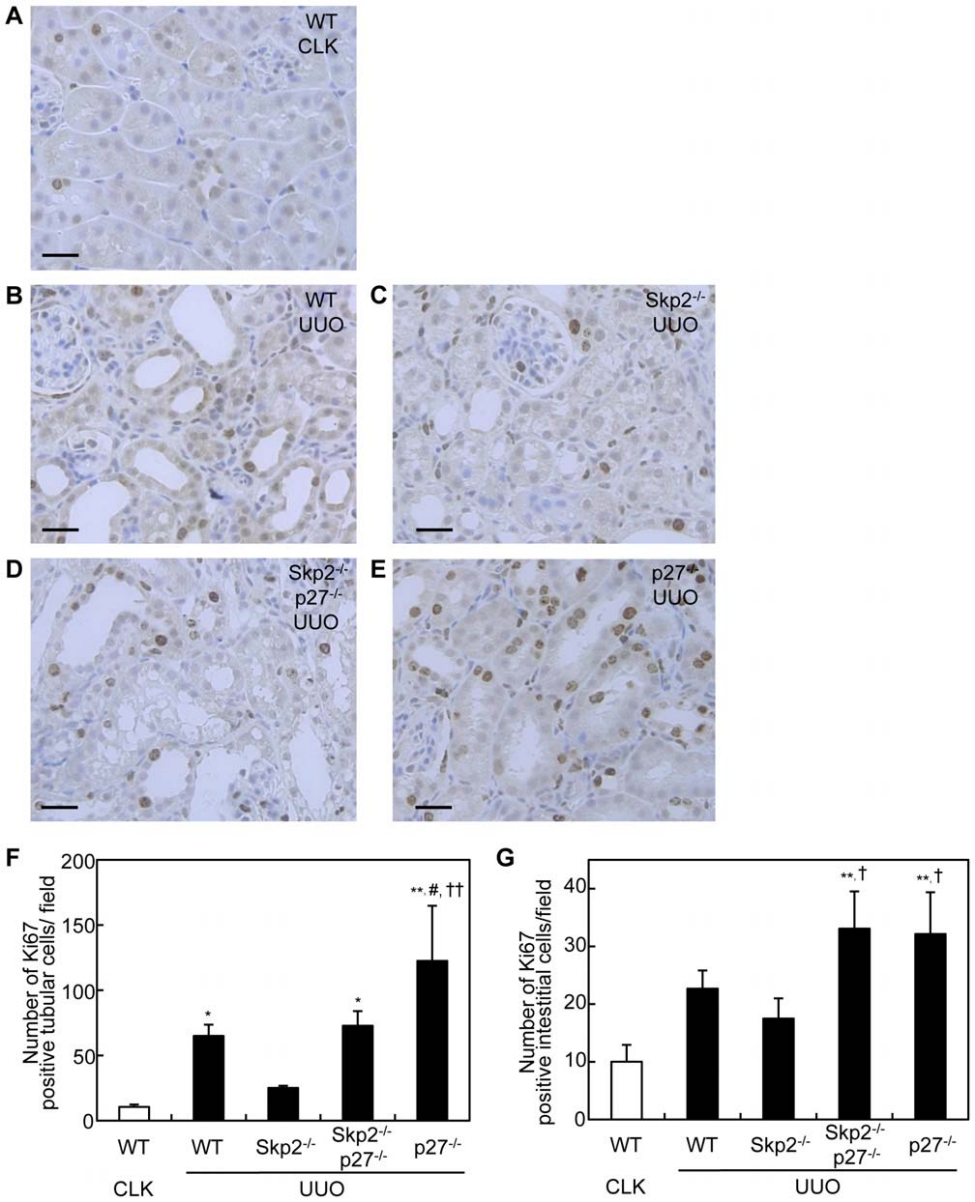
Numbers of Ki67-positive proliferative tubular epithelial and interstitial cells in UUO kidneys. (**A–E**) Sections of CLK kidneys in WT mice (**A**) and UUO kidneys in WT (**B**), Skp2^−/−^ (**C**), Skp2^−/−^p27^−/−^ (**D**) and p27^−/−^ (**E**) mice were subjected to immunostaining with an anti-Ki67 antibody, scale bars: 50 µm (**F, G**) The numbers of Ki67-positive tubular epithelial cells (**F**) and Ki67-positive interstitial cells (**G**) in the UUO kidneys were counted in the mice of each genotype. The CLK kidneys in WT mice were evaluated as controls. *P<0.05, **P<0.01 versus WT CLK kidneys, ^#^ *P*<0.05 versus WT UUO kidneys and ^†^ *P*<0.05, ^††^ *P*<0.01 versus Skp2^−/−^ UUO kidneys.

The number of Ki67-positive proliferative tubular epithelial cells per tubule was significantly increased in the UUO kidneys in WT mice, but was suppressed in Skp2^−/−^ mice. However, the suppression of tubular epithelial cell proliferation noted in the UUO kidneys in Skp2^−/−^ mice was diminished in Skp2^−/−^p27^−/−^ mice (Figure 3, A–F). A marked increase in proliferative tubular cells was observed in p27^−/−^ mice. The number of Ki67-positive interstitial cells was also increased in the UUO kidneys in WT mice compared with the CLK kidneys, and was slightly decreased in the UUO kidneys in Skp2^−/−^ mice (Figure 3, A–E and G). On the other hand, marked increases in Ki67-positive proliferative interstitial cells were observed in the UUO kidneys in Skp2^−/−^p27^−/−^ and p27^−/−^ mice.

**Figure 4:**
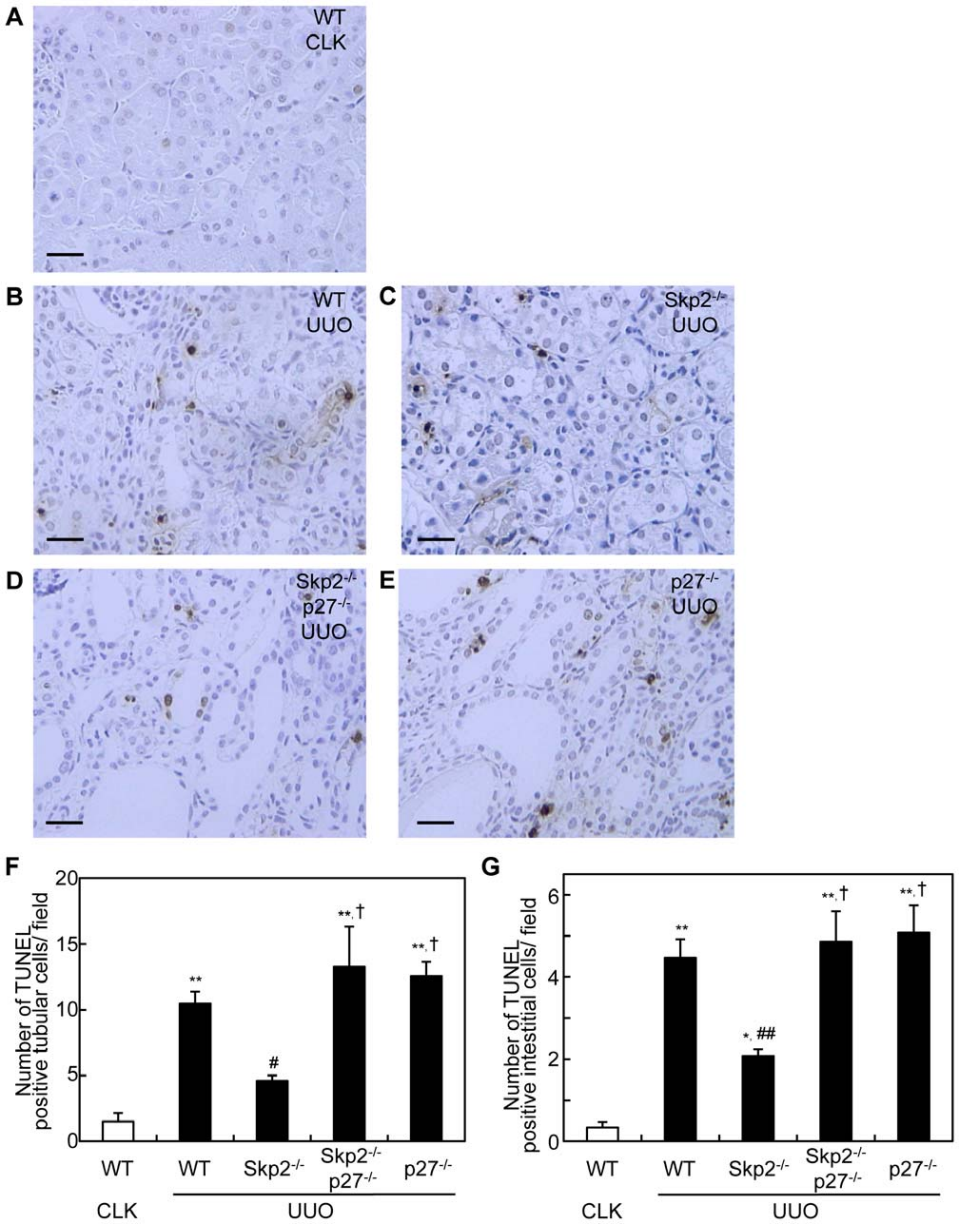
Numbers of TUNEL-positive apoptotic tubular epithelial and interstitial cells in UUO kidneys. (**A–E**) Sections from CLK kidneys in WT mice (**A**) and UUO kidneys in WT (**B**), Skp2^−/−^ (**C**), Skp2^−/−^p27^−/−^ (**D**) and p27^−/−^ (**E**) mice were subjected to TUNEL staining, scale bars: 50 µm. (**F, G**) The numbers of TUNEL-positive tubular epithelial cells (**F**) and TUNEL-positive interstitial cells (**G**) in the UUO kidneys were counted in the mice of each genotype. The CLK kidneys in WT mice were evaluated as controls. *P<0.05, **P<0.01 versus WT CLK kidneys, ^#^ *P*<0.05, ^##^ *P*<0.01 versus WT UUO kidneys and ^†^ *P*<0.01 versus Skp2^−/−^ UUO kidneys.

Compared with the CLK kidneys in WT mice, the numbers of TUNEL-positive apoptotic tubular and interstitial cells were increased in the UUO kidneys in WT mice, but were significantly fewer in Skp2^−/−^ mice. In contrast, the suppression of TUNEL-positive apoptotic tubular and interstitial cells in the UUO kidneys noted in Skp2^−/−^ mice was diminished by the additional p27-deficiency in Skp2^−/−^p27^−/−^ mice (Figure 4, A–G). The increases in the numbers of TUNEL-positive apoptotic tubular and interstitial cells were also observed in the UUO kidneys in p27^−/−^ mice.

### Diminished suppressive effect of Skp2-deficiency on progression of tubulointerstitial fibrosis in UUO kidneys in Skp2^−/−^p27^−/−^ mice

**Figure 5:**
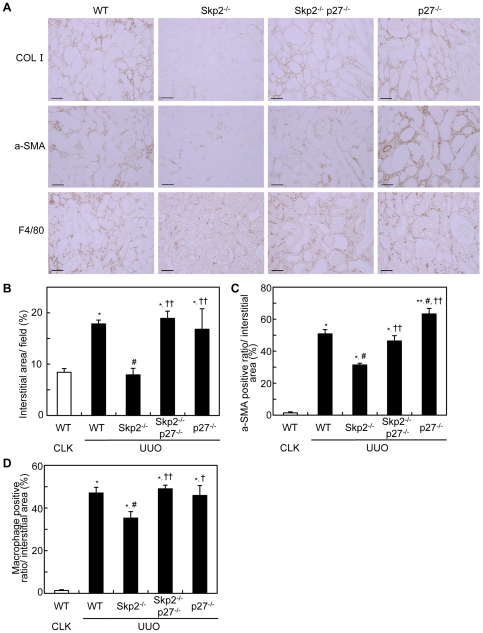
Levels of tubulointerstitial fibrotic lesions. (**A**) Representative images of UUO kidney sections in WT, Skp2^−/−^, Skp2^−/−^p27^−/−^ and p27^−/−^ mice immunostained for type I collagen (top panels), α-SMA (middle panels) and F4/80 (bottom panels) at 7 days after UUO, scale bars: 50 µm. (**B**) The ratios of the interstitial area in the UUO kidneys were quantified using the sections stained for type I collagen. (**C, D**) The intensities of the α-SMA (**C**) and F4/80 (**D**) immunostaining were quantified using Scion Image software. The CLK kidneys in WT mice were evaluated as controls. *P<0.01 versus WT CLK kidneys, ^#^ *P*<0.01 versus WT UUO kidneys and ^†^ *P*<0.05, ^††^ *P*<0.01 versus Skp2^−/−^ UUO kidneys.

Comparing with the CLK kidneys in WT mice, type I collagen-positive interstitial area was significantly increased in the UUO kidneys in WT mice, but the increment was significantly suppressed in Skp2^−/−^ mice. On the other hand, the suppression was markedly diminished by the additional p27-deficiency in the UUO kidneys in Skp2^−/−^p27^−/−^ mice (Figure 5B). Similarly, the interstitial areas positive for α-SMA and F4/80 were markedly increased in the UUO kidneys in WT mice. Although these increments were significantly suppressed in Skp2^−/−^ mice, the suppression was almost completely abolished by the additional p27-deficiency in Skp2^−/−^p27^−/−^ mice (Figure 5, A, C and D). Increases in type I collagen-positive fibrotic interstitial area, α-SMA and interstitial migration of F4/80-positive macrophages were also observed in the UUO kidneys in p27^−/−^ mice. In addition, we performed QRT-PCR to measure the mRNA expression levels of COL I, α-SMA, F4/80 and fibronectin. As shown in Figure S2, comparing with the CLK kidneys in WT mice, mRNA expression levels of type I collagen, α-SMA, F4/80 and fibronectin were increased in the UUO kidneys in WT mice, but the increments were suppressed in Skp2^−/−^ mice. On the other hand, the suppression was diminished by the additional p27-deficiency in the UUO kidneys in Skp2^−/−^p27^−/−^ mice (Figure S2). These tendencies were consistent with immunohistochemical data in Figure 5A. We also found that vimentin-positive interstitial area was significantly increased in the UUO kidneys in WT mice, but the increment was suppressed in Skp2^−/−^ mice (Figure S3). On the other hand, the suppression was markedly diminished by the additional p27-deficiency in the UUO kidneys in Skp2^−/−^p27^−/−^ mice. These results strongly suggested that the Skp2/p27 pathway may contribute to EMT in progressive UUO renal injury.

## Discussion

SCF-Skp2 is an important E3 ligase that targets several cell cycle regulatory proteins. However, it has not been fully elucidated which protein degradation targeted by Skp2 contributes to the physiological and pathological events in progressive renal injury. In Skp2^−/−^ mice, we previously reported that accumulation of p27, which had escaped from Skp2-mediated ubiquitin-proteasomal degradation, resulted in the suppression of tubular epithelial cell proliferation, tubular dilatations and tubulointerstitial fibrosis in UUO renal injury [24]. In the present study, we compared the UUO renal injury among WT, Skp2^−/−^, Skp2^−/−^p27^−/−^ and p27^−/−^ mice, and found that 1) the UUO renal injury was less severe in Skp2^−/−^ mice, in which renal accumulation of p27 was noted, 2) the amelioration of UUO renal injury noted in Skp2^−/−^ mice, was almost completely abolished, and instead rather aggravated, by the additional p27-deficiency in Skp2^−/−^p27^−/−^ double-knockout mice, and 3) the aggravation of UUO renal injury was also noted in p27^−/−^ mice. These findings suggest that the decrease in p27 targeted by Skp2 is involved in the progression of UUO renal injury.

No significant renal accumulation of p57, p130, TOB1, cyclin A or cyclin D1, which are also Skp2 targets, was observed, suggesting that these proteins are not targets for Skp2 in UUO renal injury. In contrast, increases in several other possible Skp2 targets, including p21, c-Myc, b-Myb and cyclin E, were also observed in the ameliorated UUO kidneys in Skp2^−/−^ mice, but showed slight increments compared with p27 accumulation. Because c-Myc, b-Myb and cyclin E accelerate the cell cycle and stimulate cell proliferation, it is unlikely that their increases are involved in the suppression of tubular and interstitial cell proliferation in UUO renal injury in Skp2^−/−^ mice. However, the role of the moderately increased p21, another CDKI possibly targeted by Skp2, in the suppression of UUO renal injury in Skp2^−/−^ mice remains to be clarified. Since it has been reported that p21 regulates interstitial cells in UUO kidneys [23], the decrease in p21 targeted by Skp2 may be involved in the progression of interstitial cell proliferation in UUO kidneys. Further studies using Skp2^−/−^p21^−/−^ double-knockout mice are needed to clarify the role of p21 in UUO renal injury.

p27 deletion not only abolished the protective effects of Skp2^−/−^ genotype, but also makes the renal lesion worse compared to the wild type. p27 deletion may promote unscheduled activations of various cyclin-dependent kinases. Thereby renal epithelial cell growth and following tubular dilation induced by renal obstruction may be irregularly enhanced in p27^−/−^ mice. In the Skp2^−/−^ (Figure 1) and Skp2^−/−^p27^−/−^ mice (data not shown), another Skp2 targets such as p21 and c-Myc are accumulated. c-Myc plays roles for promotion of cell growth and apoptosis, whereas p27 and p21 are negative regulators in cell proliferation. The increased c-Myc may play opposite roles against p27 and p21 for tubular epithelial cell proliferation. Moreover, it has been reported that p21 inhibits c-Myc expression [31]. c-Myc promotes Skp2-mediated degradation of p27 [32]. Therefore, Skp2 target cell cycle regulators are functionally related each other. Skp2 deletion may induce these unbalances of cell cycle regulators not only directly but also indirectly, thereby the cell proliferation and apoptosis were abrogated in the UUO kidney.

In addition to the diminished amelioration of tubular and interstitial cell proliferation, aggravation of interstitial fibrosis composed of increased deposition of type I collagen, interstitial expression of α-SMA, and infiltration of macrophages was noted in the UUO kidneys in Skp2^−/−^p27^−/−^ and p27^−/−^ mice. These findings suggest that the regulation of p27 by Skp2 is involved in the development of tubulointerstitial fibrosis. It has been reported that type II epithelial-mesenchymal transdifferentiation (EMT) occurs in progressive nephropathies to differentiate myofibroblasts from renal epithelial cells [33]. The expression levels of α-SMA, fibronectin and vimentin are closely associated with type II EMT as typical markers for myofibroblasts [33]–[36]. Our data suggest that the Skp2/p27 pathway may contribute to EMT in progressive UUO renal injury, although further studies are required.

Talking all our findings into consideration, it is suggested that degradation of p27 targeted by Skp2 accelerated the progression of renal tubular epithelial cell proliferation, tubular dilation and tubulointerstitial fibrosis in UUO renal injury. Therefore, we conclude that p27 is a main target for Skp2 and that degradation of p27 by Skp2 is required for the progression of renal injury mediated by UUO. Based on this, inhibitors of SCF-Skp2/Cks1 E3 ligase that specifically promote p27 degradation may be effective for the treatment of progressive nephropathy. Further studies are required to examine this possibility.

## Methods

### Experimental animals

The generation of Skp2^−/−^ mice [13] and p27^−/−^ mice [37] were signed by Nakayama et al. To obtain WT [Skp2^+/+^p27^+/+^], Skp2^−/−^, Skp2^−/−^p27^−/−^ and p27^−/−^ offspring, heterozygous Skp2^+/−^p27^+/−^ mice were mated. The mice included in this study were 6-week-old male mice weighing 20–25 g. The mice were allowed free access to food and water, and were maintained under a 12-h/12-h light-dark cycle. We confirmed the WT and disrupted alleles of Skp2 and p27 by PCR. The mice were treated according to protocols approved by the Hamamatsu University School of Medicine Animal Care Committees at the Center Animal Care facility.

### Experimental design

Ureteral obstruction was achieved by ligating the left ureter with 3-0 silk through a left lateral incision. WT, Skp2^−/−^, Skp2^−/−^p27^−/−^ and p27^−/−^ mice (n = 4–5 per group) were sacrificed at 7 days after the operation. The obstructed (UUO) and non-obstructed contralateral (CLK) kidneys were harvested from each mouse and subjected to the analyses described below. The experimental protocol was approved by the Ethics Review Committee for Animal Experimentation of Hamamatsu University School of Medicine.

### Immunoblot analysis

Whole kidney tissues were dissolved in RIPA buffer (25 mmol/L Tris-HCl pH 7.4, 150 mmol/L NaCl, 0.1% SDS, 0.5% Triton-X100, 0.5% sodium deoxycholate) containing protease inhibitors (150 mg/ml PMSF, 5 mg/ml aprotinin, 5 mg/ml pepstatin, 5 mg/ml leupeptin, 5 mg/ml E-64) at 4°C. After incubation for 30 min, the lysates were centrifuged at 13000×*g* for 15 min at 4°C. The protein concentrations of the lysates were measured using a protein assay reagent (Bio-Rad Laboratories, Hercules, CA). Soluble lysates were boiled with 4×SDS sample buffer (250 mmol/L Tris-HCl pH 6.8, 12% SDS, 40% glycerol, 20% 2-mercaptoethanol, 1% bromophenol blue) for 8 min. Equal amounts of proteins were loaded and separated by SDS-PAGE. The separated proteins were transferred to polyvinylidene difluoride membranes, followed by immunoblotting with the following antibodies: mouse monoclonal anti-p27 and anti-p130 (BD Transduction Laboratories, San Jose, CA); anti-p21 (Santa Cruz Biotechnology, Santa Cruz, CA); anti-Myc (Cell Signaling Technology, Danvers, MA); anti-α-tubulin (Sigma, St. Louis, MO); rabbit monoclonal anti-p57 (Abcam, Cambridge, UK); rabbit polyclonal anti-TOB (Abcam); anti-b-Myb, anti-cyclin A, anti-cyclin D1 and anti-cyclin E (Santa Cruz Biotechnology). α-tubulin was evaluated as an internal control. The antibody-bound proteins were visualized using an enhanced chemiluminescence system (Perkin Elmer, Wellesley, MA).

### Histopathological and immunohistochemical analyses

Kidney tissues were fixed in 4% paraformaldehyde in PBS and embedded in paraffin. Tissue sections (3-µm thickness) were rehydrated and subjected to Masson's trichrome staining for histopathological analysis. The levels of fibrotic tubulointerstitial lesions were graded semiquantitatively as follows: 0, absent (0%); 1, weak (≤10%); 2, mild (>10 to ≤30%); 3, moderate (>30 to ≤50%); 4, strong (>50%) in twenty randomly selected non-overlapping renal cortical fields at ×400 magnification. The percentage of dilated tubules per total tubules, the number of renal tubular epithelial cells in a tubule which was obtained as the number of tubular epithelial cells divided by the number of tubules, and the number of interstitial cells were counted in 10 randomly selected non-overlapping renal cortical fields at ×400 magnification. The immunoreactivities for Ki67, type I collagen, α-smooth muscle actin (α-SMA) and F4/80, a marker protein for macrophages, were determined using a Histofine SAB-PO kit (Nichirei, Tokyo, Japan) as a standard biotin-streptavidin-peroxidase method as described previously [24]. The primary antibodies were as follows: rabbit polyclonal anti-human Ki67 (Novocastra Laboratories, Newcastle-upon-Tyne, UK); anti-mouse type I collagen (Abcam); mouse monoclonal anti-human α-SMA (DAKO, Hamburg, Germany); and rat anti-mouse F4/80 (Serotec, Oxford, UK). The secondary antibodies were affinity-purified biotinylated goat anti-mouse or anti-rabbit immunoglobulin, and peroxidase-conjugated anti-rat immunoglobulin (Nichirei, Tokyo, Japan). The kidney sections were lightly counterstained with hematoxylin. The numbers of Ki67-positive tubular epithelial and interstitial cells were counted in 10 randomly selected non-overlapping renal cortical fields at ×400 magnification and the mean values were obtained. The type I collagen-positive interstitial area and the areas positive for α-SMA and F4/80 were quantified in 10 randomly selected non-overlapping fields at ×400 magnification using Scion Image software (Scion Corp., Frederick, MD). We set a threshold to automatically compute the positive areas for each stain and computed the ratio of the positive areas to the whole interstitial area.

### Evaluation of apoptosis

Deoxynucleotidyl transferase-mediated dUTP nick end labeling (TUNEL) was performed to detect apoptotic cell death using an ApopTag Plus Peroxidase *In Situ* Apoptosis Detection Kit (Chemicon, Temecula, CA). For each kidney, the number of apoptotic tubular epithelial and interstitial cells in 10 non-overlapping renal cortical fields was counted under ×400 magnification. The number of TUNEL-positive nuclei was averaged for each field.

### Statistical analysis

Statistical analysis was performed using computer-assisted software (StatView 5.0; SAS Institute Inc.). All values are given as means ± SEM. Differences between groups were examined for statistical significance by analysis of variance. When a significant difference was found between two groups, a further statistical analysis was performed using the Bonferroni–Dunn test. Values of *P*<0.05 were considered to indicate statistical significance.

## Supporting Information

Figure S1 **The expression of Skp2 and/or p27 in WT, Skp2^−/−^, Skp2^−/−^p27^−/−^ and p27^−/−^ mice.** The genotypes of the WT, Skp2^−/−^, Skp2^−/−^p27^−/−^ and p27^−/−^ mice were confirmed by PCR (**A**). The PCR primer sequences of Skp2 were sense 5′-CAGACCCTGACGCACCTCACG-3′ and antisense 5′-TTCTGACGCCCCGTTGCCTGCT-3′for WT, 5′-GGTGGATGTGGAATGTGTGCGAGGC-3′ for knockout allele; and of p27 were sense 5′-CGTGGGGTGTAGAATACTCCTTGT-3′ and antisense 5′-GATACGACCGTCCCTATCCTTTG-3′ for WT, 5′-TGCTAAAGCGCATGCTCCAGACTG-3′ for KO. Primers for glyceraldehyde-3-phosphate dehydrogenase (GAPDH), used as an internal control, were sense 5′-TGCACCACCAACTGCTTAG-3′ and antisense 5′-GATGCAGGGATGATGTTC-3′. The protein level of p27 in CLK (C) and UUO (U) kidneys in each genotype mouse (**B**). α-tubulin was used an internal control.
(TIF)

Figure S2 **mRNA expression of UUO renal injury.** mRNA level of COL I (**A**), α-SMA (**B**), F4/80 (**C**) and Fibronectin (**D**) measured by quantitative RT-PCR from CLK in WT mouse, and UUO kidneys in WT, Skp2^−/−^, Skp2^−/−^p27^−/−^ and p27^−/−^ mice. Total RNA was extracted from whole kidney tissue using the Isogen (Wako, Osaka, Japan) according to the manufacturer's instructions. Reverse transcription of the RNA was performed using the SuperScript First-Strand Synthsis System for RT-PCR kit (Invitrogen, Carlsbad, CA) with 2.5 µg of total RNA. The resulting cDNA was subjected to real-time PCR using the Roter-Gene 3000 System (Corbett Research, Mortlake, Australia) for amplification and online quantification. All PCR experiments were performed using a QuantiTect SYBR Green PCR kit purchased from TAKARA (TAKARA, Shiga, Japan). The PCR-primer sequences for COL I were sense 5′-AGAGCATGACCGATGGATTCC-3′ and antisense 5′-TTGCCAGTCTGCTGGTCCATG-3′ for α-SMA were sense 5′- ACTGGGACGACATGGAAAAG-3′ and antisense 5′-CATCTCCAGAGTCCAGCACA-3′: for F4/80 were sense 5′-GATGGGGGATGACCACACTT -3′ and antisense 5′-TTCAGGGCAAACGTCTCG-3′: for Fibronectin were sense 5′-ACGGTTTCCCATTACGCCAT-3′ and antisense 5′-CTTTCCATTCCCGAGGCAT-3′. GAPDH was evaluated as an internal control. The amount of COL I, α-SMA, F4/80 and Fibronectin mRNA was normalized for GAPDH mRNA in each sample. The CLK kidneys in WT mice were evaluated as controls. ^*^ *P*<0.05, ^**^ *P*<0.005 versus WT CLK kidneys, ^#^ *P*<0.05 versus WT UUO and ^†^ *P*<0.05 versus Skp2^−/−^ UUO.
(TIF)

Figure S3 **The immunoreactivity for Vimentin in UUO kidneys.** Representitive images of immunohistochemical staining for Vimentin from WT CLK and UUO kidneys in each genotype mouse. The primary antibody was rabbit polyclonal anti-human Vimentin (Santa Cruz), scale bars: 50 µm.
(TIF)
