# Supplementary figures and images for: The Amelioration of Renal Damage in Skp2-Deficient Mice Canceled by p27 Kip1 Deficiency in Skp2−/− p27−/− Mice
Source: PLoS One. 2012 Apr 27;7(4):e36249. doi: 10.1371/journal.pone.0036249 (PMC3338689; doi:10.1371/journal.pone.0036249)

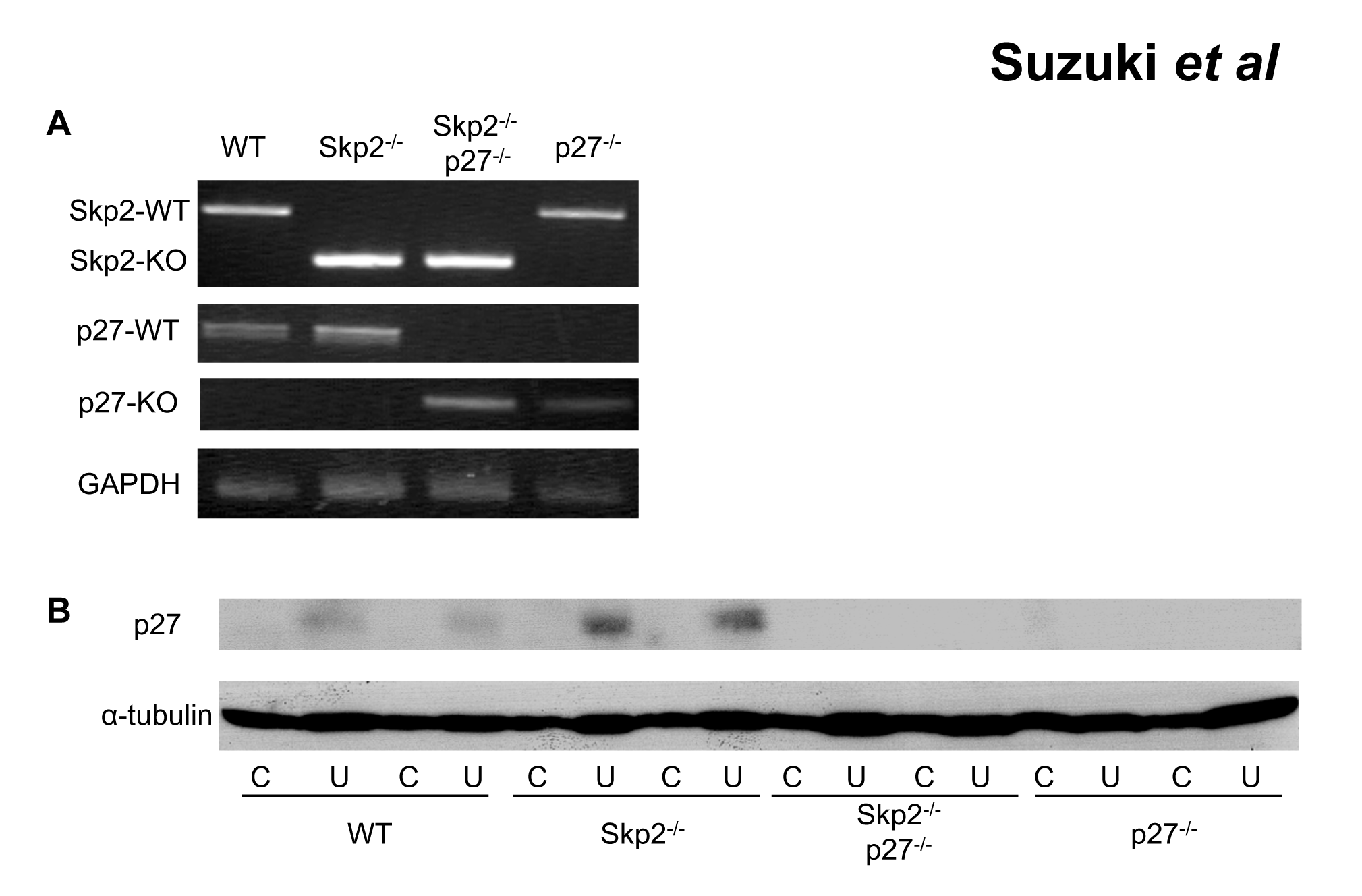

Supplement: Figure S1 — The expression of Skp2 and/or p27 in WT, Skp2−/−, Skp2−/−p27−/− and p27−/− mice. The genotypes of the WT, Skp2−/−, Skp2−/−p27−/− and p27−/− mice were confirmed by PCR (A). The PCR primer sequences of Skp2 were sense 5′-CAGACCCTGACGCACCTCACG-3′ and antisense 5′-TTCTGACGCCCCGTTGCCTGCT-3′for WT, 5′-GGTGGATGTGGAATGTGTGCGAGGC-3′ for knockout allele; and of p27 were sense 5′-CGTGGGGTGTAGAATACTCCTTGT-3′ and antisense 5′-GATACGACCGTCCCTATCCTTTG-3′ for WT, 5′-TGCTAAAGCGCATGCTCCAGACTG-3′ for KO. Primers for glyceraldehyde-3-phosphate dehydrogenase (GAPDH), used as an internal control, were sense 5′-TGCACCACCAACTGCTTAG-3′ and antisense 5′-GATGCAGGGATGATGTTC-3′. The protein level of p27 in CLK (C) and UUO (U) kidneys in each genotype mouse (B). α-tubulin was used an internal control. (TIF) [file pone.0036249.s001.tif]

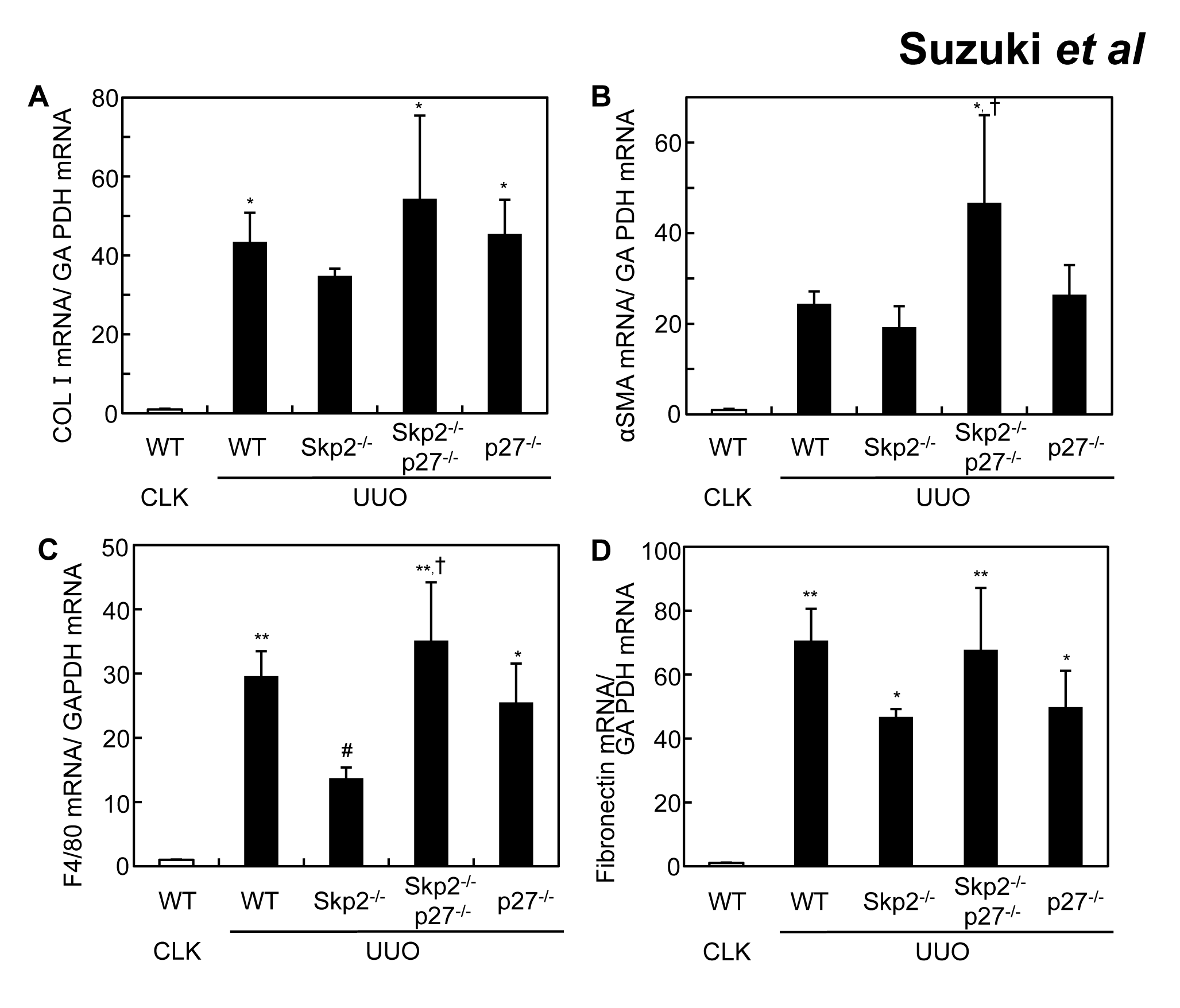

Supplement: Figure S2 — mRNA expression of UUO renal injury. mRNA level of COL I (A), α-SMA (B), F4/80 (C) and Fibronectin (D) measured by quantitative RT-PCR from CLK in WT mouse, and UUO kidneys in WT, Skp2−/−, Skp2−/−p27−/− and p27−/− mice. Total RNA was extracted from whole kidney tissue using the Isogen (Wako, Osaka, Japan) according to the manufacturer's instructions. Reverse transcription of the RNA was performed using the SuperScript First-Strand Synthsis System for RT-PCR kit (Invitrogen, Carlsbad, CA) with 2.5 µg of total RNA. The resulting cDNA was subjected to real-time PCR using the Roter-Gene 3000 System (Corbett Research, Mortlake, Australia) for amplification and online quantification. All PCR experiments were performed using a QuantiTect SYBR Green PCR kit purchased from TAKARA (TAKARA, Shiga, Japan). The PCR-primer sequences for COL I were sense 5′-AGAGCATGACCGATGGATTCC-3′ and antisense 5′-TTGCCAGTCTGCTGGTCCATG-3′ for α-SMA were sense 5′- ACTGGGACGACATGGAAAAG-3′ and antisense 5′-CATCTCCAGAGTCCAGCACA-3′: for F4/80 were sense 5′-GATGGGGGATGACCACACTT -3′ and antisense 5′-TTCAGGGCAAACGTCTCG-3′: for Fibronectin were sense 5′-ACGGTTTCCCATTACGCCAT-3′ and antisense 5′-CTTTCCATTCCCGAGGCAT-3′. GAPDH was evaluated as an internal control. The amount of COL I, α-SMA, F4/80 and Fibronectin mRNA was normalized for GAPDH mRNA in each sample. The CLK kidneys in WT mice were evaluated as controls. * P<0.05, ** P<0.005 versus WT CLK kidneys, # P<0.05 versus WT UUO and † P<0.05 versus Skp2−/− UUO. (TIF) [file pone.0036249.s002.tif]

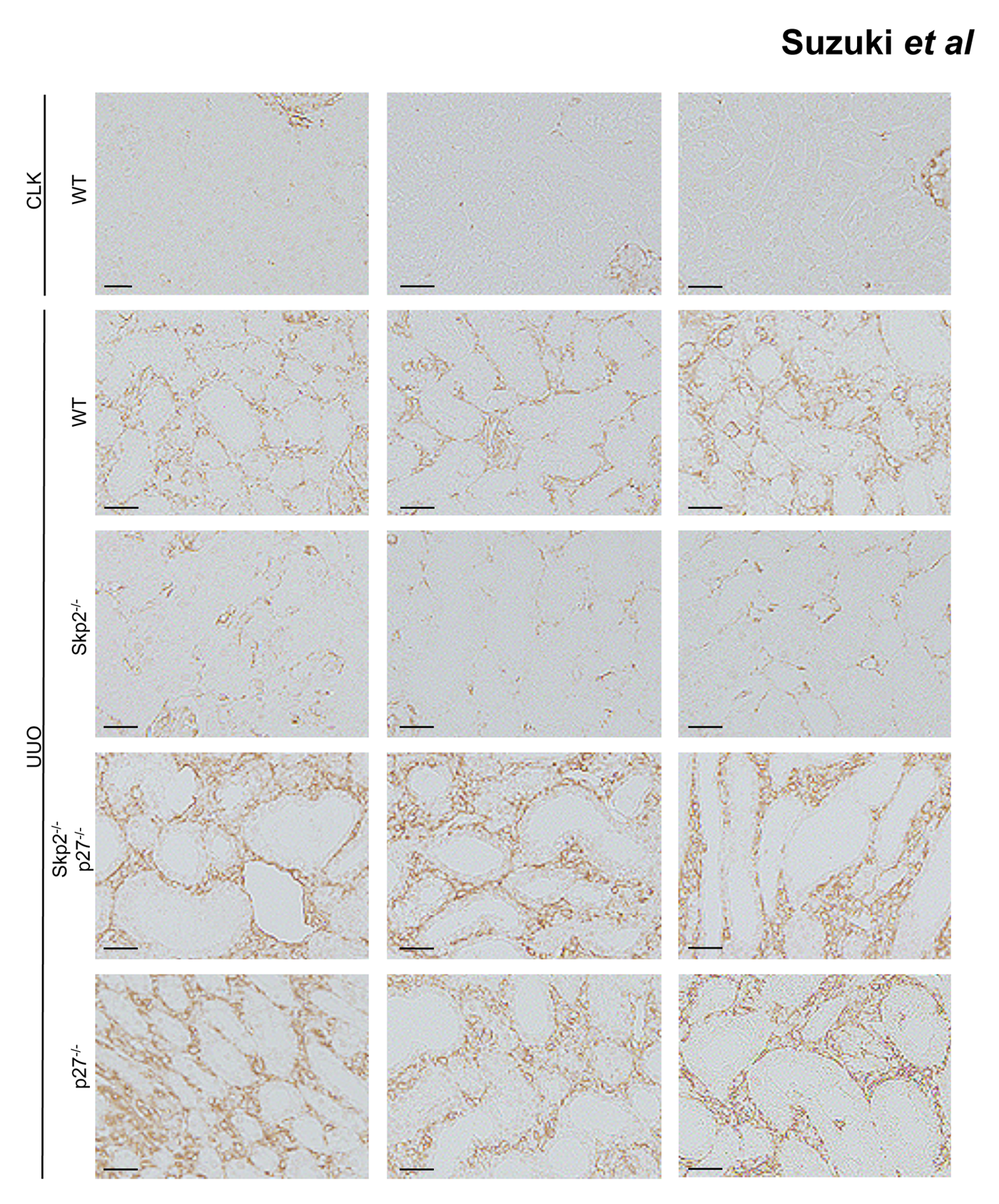

Supplement: Figure S3 — The immunoreactivity for Vimentin in UUO kidneys. Representitive images of immunohistochemical staining for Vimentin from WT CLK and UUO kidneys in each genotype mouse. The primary antibody was rabbit polyclonal anti-human Vimentin (Santa Cruz), scale bars: 50 µm. (TIF) [file pone.0036249.s003.tif]
